# Tarlov cyst: Case report and review of literature

**DOI:** 10.4103/0019-5413.37007

**Published:** 2007

**Authors:** Bhagwat Prashad, Anil K Jain, Ish K Dhammi

**Affiliations:** University College of Medical Sciences and Guru Teg Bahadur Hospital, Shahadara, Delhi, India

**Keywords:** Low back pain, sacral perineural cyst, sciatica, tarlov cyst

## Abstract

We describe a case of sacral perineural cyst presenting with complaints of low back pain with neurological claudication. The patient was treated by laminectomy and excision of the cyst. Tarlov cysts (sacral perineural cysts) are nerve root cysts found most commonly in the sacral roots, arising between the covering layer of the perineurium and the endoneurium near the dorsal root ganglion. The incidence of Tarlov cysts is 5% and most of them are asymptomatic, usually detected as incidental findings on MRI. Symptomatic Tarlov cysts are extremely rare, commonly presenting as sacral or lumbar pain syndromes, sciatica or rarely as cauda equina syndrome. Tarlov cysts should be considered in the differential diagnosis of patients presenting with these complaints.

Tarlov cysts were first described in 1938 as an incidental finding at autopsy.^1^ Tarlov described a case of symptomatic perineural cyst and recommended its removal. Since then a few cases have been reported in the literature.^2^–^4^

Paulsen reported the incidence of Tarlov cysts as 4.6% in back pain patients (n=500). Only 1% of back pain patients (n=500) were symptomatic.^4^ The patient may present as low back pain, sciatica, coccydynia or cauda equina syndrome. The cysts are usually diagnosed on MRI, which reveals the lesion arising from the sacral nerve root near the dorsal root ganglion.^5^

Tarlov advised extensive surgery with sacral laminectomy and excision of the cyst along with the nerve root.^6^ Paulsen reported CT-guided percutaneous aspiration of these perineural cysts for relief of sciatica.^4^ Recently, microsurgical excision of the cyst has been advocated, combined with duraplasty or plication of the cyst wall.^7^

We report a case of symptomatic Tarlov cyst presenting as back pain, to increase the awareness of this rare entity in the orthopedic community.

## CASE REPORT

A 29-year-old female presented with right thigh pain off and on for nine months. The pain was not associated with specific time, posture or activity and it used to get relieved by non steroidal antiinflammatory drugs (NSAID). Clinical examination at this stage did not reveal any findings at spine, bilateral hips and left thigh.

For last three months, the intensity and duration of pain had increased, which was now unrelieved by taking NSAID. The pain had progressed to the lower back and bilateral upper thigh up to the ankle. The pain was aggravated by activity and prolonged standing and was more bothersome in the evening. She used to get up in the middle of the night with pain. Later the patient started having rest pain as well. Examination showed no spinal tenderness. Straight leg raising was 50° on the right side and normal on the left side. There was mild blunting of sensations along the S1 and S2 dermatome on the right side, no motor deficit in both lower limbs.

**Figure 1:**
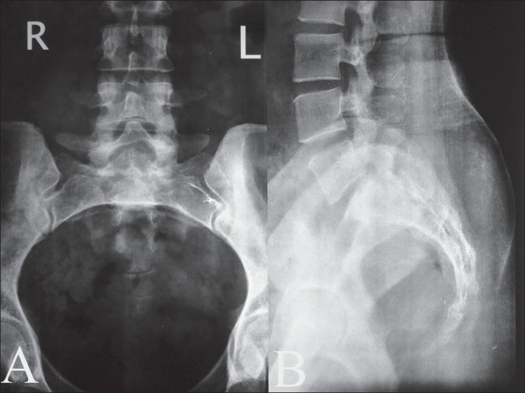
X-ray of lumbosacral spine antero posterior (A) and lateral (B) view of the patient showing no obvious bony changes

**Figure 2:**
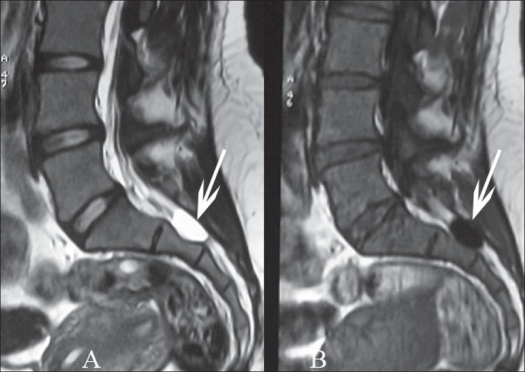
MRI mid segittal T_2_WI (A) and T_1_WI (B) imaging showing a fluid-filled cystic lesion, measuring 2cm in diameter, sitting opposite the second sacral vertebra

X-ray of the lumbosacral spine did not reveal any abnormality [Figure 1]. The MRI of the spine revealed fluid-filled cystic lesion, arising from the second sacral nerve root on the right side and measuring 2cm in diameter [Figure 2].

**Figure 3(A-D):**
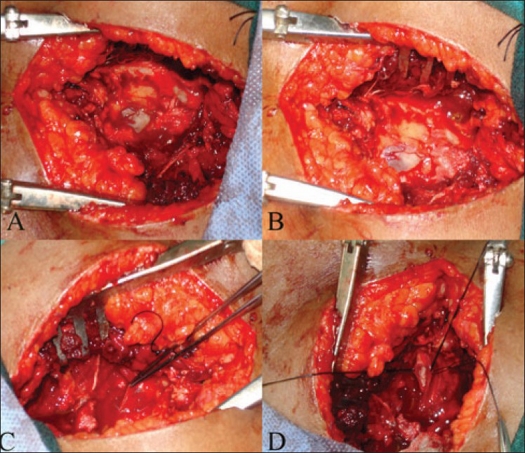
Peroperative photographs of the case showing Sacral laminectomy (A) exposed cyst (B). After excising the cyst (C), Plication of cyst wall (D)

The patient was taken for sacral laminectomy, excision of the cyst and plication of the cyst wall, while retaining the nerve root [Figure 3]. Histopathological examination of the cyst wall showed nerve cells, which confirmed the diagnosis of Tarlov cyst.

**Figure 4:**
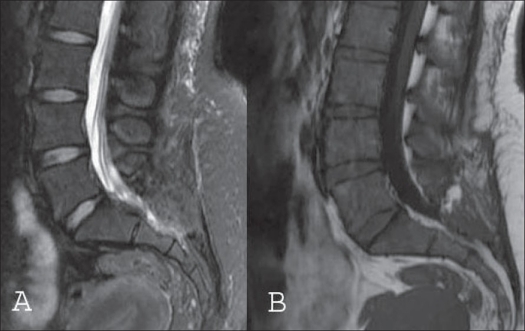
Mid-segittal T_2_WI (A) and T_1_WI (B) taken at nine months postoperative followup did not show an evidence of recurrence of the cyst

Patient appreciated relief of pain immediately after the surgery. Postoperative period was uneventful and the patient made prompt recovery. On nine months followup, the patient had no pain in lower limbs and back. The patient is back at her job and is asymptomatic. Postoperative MRI taken at nine months [Figure 4] did not show any evidence of recurrence of the cyst.

## DISCUSSION

Tarlov cysts are rare causes of low back pain. They are more common in females.^4^,^7^ Clinical presentation of Tarlov cysts is variable. The cysts may cause local and/or radicular pain. The dominant syndrome is referable to the caudal nerve roots, either sciatica, sacral or buttocks pain, vaginal or penile paraesthesia or sensory changes over the buttocks, perineal area and lower extremity. Depending on their location, size and relationship to the nerve roots, they may cause sensory disturbances or motor deficits to the point of bladder dysfunction. Tenderness on firm pressure over the sacrum may be present. Commonly, the symptomatology is intermittent at its onset and is most frequently exacerbated by standing, walking and coughing. Bed rest alleviates the discomfort.^8^

Plain X-rays are usually normal. However, they may reveal characteristic bone erosion of the spinal canal or anterior or posterior neural foramina.^9^ A CT scan can demonstrate cystic masses isodense with CSF located at the foramina. Bony changes may also be present.^10^ An MRI gives a much better soft tissue contrast and is currently the investigation of choice for perineural cysts. The cysts demonstrate low signal on T-1 weighted images and high signal on T-2 weighted images, similar to CSF.^5^ Myelography showing the filling of the meningocele sac 1h after injection of contrast medium is highly suggestive of a perineural cyst.^11^

Microscopic features of the cyst were described by Tarlov. The early stage in cyst formation is that of a space between the arachnoid which covers the root or the perineurium and the outer layer of the pial cover of the root or the endoneurium. It usually begins in one portion of the circumference of the perineural space, the larger cysts compressing the nerve root to one side. The cyst occupies the posterior root abutting the proximal portion of the dorsal ganglion. Its main part is bordered by reticulum or by nerve fibers.^1^

The pathogenesis of perineural cysts is uncertain. Tarlov felt that hemorrhage into the subarachnoid space caused accumulations of red cells which impeded the drainage of the veins in the perineurium and epineurium, leading to rupture with subsequent cyst formation. Four out of the seven patients in Tarlov's 1970 article had a history of trauma.^8^ Schreiber and Haddad also supported this posttraumatic cause of cyst formation.^12^ Because many of the patients with perineural cyst in their series did not have histories of trauma, Fortuna *et al*. believed that the perineural cysts were congenital, caused by arachnoidal proliferations within the root sleeve.^13^

There is no consensus on a single method of treatment. Various methods have been advocated. Tarlov advised that symptomatic, single perineural cysts should be completely excised together with the posterior root and ganglion from which they arise.^8^ Paulsen reported CT-guided percutaneous aspiration of these perineural cysts in two patients for the relief of sciatica caused by compression.^4^ According to Caspar microsurgical excision of the cyst combined with duraplasty or plication of the cyst wall is an effective and safe treatment of symptomatic sacral cysts. The parent nerve root is always left intact.^7^

Tarlov cysts are a documented cause of sacral radiculopathy and other radicular pain syndromes. They must be considered in the differential diagnosis of patients presenting with these clinical presentations and appropriately treated by cyst excision.
